# Development and validation of a CT-based habitat radiomics model for predicting pathological grading in non-small cell lung cancer

**DOI:** 10.3389/fmed.2026.1722634

**Published:** 2026-02-12

**Authors:** Dexuan Xie, Chongyang Sun, Ming Xue, Xigang Xiao

**Affiliations:** Department of Radiology, First Affiliated Hospital of Harbin Medical University, Harbin, China

**Keywords:** computed tomography, grading, habitat, non-small cell lung cancer, radiomics

## Abstract

**Objective:**

To develop and validate models for predicting pathological grading of non-small cell lung cancer (NSCLC) using habitat radiomics and clinical semantic features.

**Materials and methods:**

In this retrospective study of 800 NSCLC patients, a whole tumor volume (WTV) was delineated by applying a 3 mm expansion to the gross tumor volume (GTV) on non-contrast CT scans. Habitat subregions within the WTV were identified using K-means clustering. A two-step binary classification model was constructed to predict pathological grades: Model-1 distinguished Grade 3 from combined Grades 1–2, and Model-2 further differentiated Grade 1 from Grade 2. Predictive models were built with logistic regression based on four distinct feature sets: WTV radiomics (Clf WVOI), habitat radiomics (Clf Habitats), clinical features (Clf Clinical), and a combined feature set (Clf Total).

**Results:**

In both Model-1 and Model-2, the classification performance of Clf Habitats was generally superior to that of Clf WVOI and Clf Clinical, achieving an AUC of 0.89 and 0.87, specificity of 0.73 for both models, and BACC of 0.78 and 0.79, respectively, on the test set. The combined model, Clf Total, achieved the best predictive performance on the test set, with AUC values of 0.91 and 0.88, specificity of 0.84 and 0.77, and BACC of 0.82 and 0.81.

**Conclusion:**

Habitat radiomics significantly improves NSCLC pathological grading. The multimodal model offers robust performance and high specificity, aiding personalized treatment planning.

## Graphical Abstract

**Figure:**
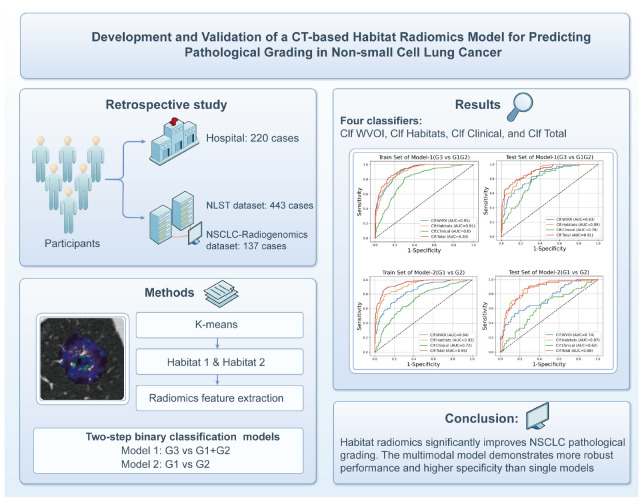
Left: Schematic of the multicenter retrospective study and methodological pipeline, including K means–based habitat segmentation, radiomics feature extraction, and a two step classification framework. Right: Comparison of four classifiers shows that integrating habitat radiomics improves prediction of NSCLC pathological grading.

## 1 Introduction

Lung cancer is a major global health challenge. According to the International Agency for Research on Cancer (IARC) statistics, it constitutes 12.4% of newly diagnosed cancers and is the leading cause of cancer mortality, accounting for 18.7% of cancer deaths (1). NSCLC, the predominant subtype representing 80–85% of cases, has a 5-year survival rate below 20%. Tumor grading is biologically and prognostically critical: high-grade tumors are associated with significantly worse outcomes, including higher risks of metastasis and recurrence (2, 3). Accurate grading is essential for prognostic stratification and treatment planning. Percutaneous biopsy and frozen section analysis are diagnostic cornerstones but have limitations: tumor spatial heterogeneity causes sampling bias and unreliable assessments (4); invasive procedures risk pneumothorax and hemorrhage; and technical constraints limit utility in small nodules (5).

Computed tomography (CT) is one of the principal imaging modalities for lung cancer. Nonetheless, reliance solely on radiologists’ subjective interpretation of CT morphological features is insufficient for precise tumor grading. Radiomics enables the quantification of high-dimensional imaging features that are imperceptible to human visual inspection. However, conventional radiomics typically focuses on the entire tumor region for feature extraction, thereby failing to accurately characterize the complex spatial heterogeneity within the tumor. Extensive research has shown that although traditional radiomics models exhibit satisfactory performance in tumor-related predictive tasks, their inability to adequately account for heterogeneity across distinct intratumoral regions constrains further improvements in predictive accuracy (6, 7). Habitat radiomics is an analytical approach derived from conventional radiomics. It applies unsupervised machine learning algorithms to partition tumors into distinct subregions. This method effectively characterizes intratumoral heterogeneity and reveals subregion-specific microenvironmental and molecular phenotypes. Many clinical studies have demonstrated that this approach offers significant advantages in oncology by providing novel radiomic biomarkers that support the development of personalized therapeutic strategies (8–10).

Despite numerous studies establishing predictive models for pathological grading of NSCLC using radiomics, there remain several limitations: Most studies have predominantly focused on adenocarcinoma, with limited inclusion of subtypes such as squamous cell carcinoma (SCC) and large cell carcinoma (LCC); many studies have focused on identifying low-differentiated groups, without further distinguishing between moderately and highly differentiated groups; moreover, existing models have not sufficiently taken into account the spatial heterogeneity of tumors, which is a crucial factor (11–13). These factors collectively limit the generalizability and practical clinical utility of the models in real settings.

This study aims to develop and validate a habitat radiomics model using non-enhanced CT, with the goal of integrating the extracted features with key clinical semantic features into a combined predictive model for NSCLC pathological grading.

## 2 Materials and methods

This retrospective study was approved by the Ethics Committee of the First Affiliated Hospital of Harbin Medical University. Informed consent was waived due to the retrospective nature of the research.

### 2.1 Participants

The data for this study were obtained from a hospital cohort and two public datasets. The hospital cohort included 220 patients who underwent surgical resection at the First Affiliated Hospital of Harbin Medical University between May 2021 and December 2024. Data from two independent public datasets were incorporated: 443 cases from the National Lung Screening Trial (NLST) database^1^ and 137 cases from the NSCLC-Radiogenomics dataset.^2^

### 2.2 Inclusion and exclusion criteria

Inclusion criteria were as follows: (1) Histopathologically confirmed NSCLC with a definitive pathological grade; (2) No history of any anticancer treatment prior to baseline CT imaging; (3) Availability of complete clinical data, including gender, age, and smoking history.

Exclusion criteria were as follows: (1) Absence of a definitive pathological grade; (2) Presence of significant artifacts on CT images; (3) An interval > 1 month between baseline CT imaging and surgical resection.

**FIGURE 1:**
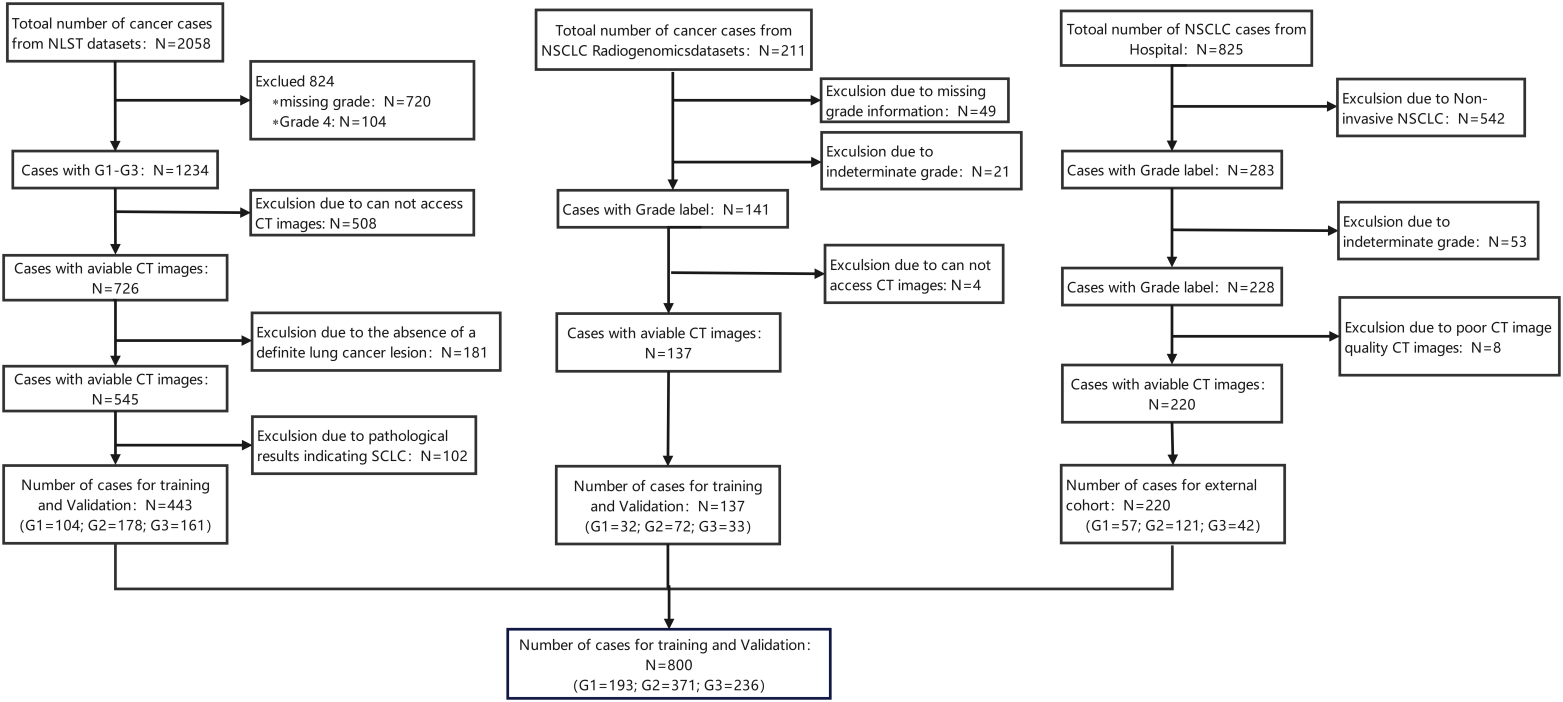
Screening flowchart of study subjects.

Patients from the three cohorts were screened according to the above criteria, and the overall selection process is illustrated in Figure 1.

Patient demographics from the NLST, NSCLC Radiogenomics, and Hospital cohorts are summarized in Table 1.

**TABLE 1:** Demographics and tumor characteristics of three cohorts.

Characteristics	Subjects	Cohorts		
		NLST	NSCLC Radiogenomics	Hospital
Age (years, mean ± SD)	800	63.75 ± 5.36	68.60 ± 9.14	60.04 ± 9.33
**Gender, n (%)**				
Female	354	188 (42.44 %)	39 (28.47 %)	127 (57.73 %)
Male	446	255 (57.56 %)	98 (71.53 %)	93 (42.27 %)
**Grading of tumor, n (%)**				
Grade1	193	104 (23.48 %)	32 (23.36 %)	57 (25.91 %)
Grade2	371	178 (40.18 %)	72 (52.55 %)	121 (55.00 %)
Grade3	236	161 (36.34 %)	33 (24.09 %)	42 (19.09 %)
**Histology, n (%)**				
ADC	620	312 (70.43 %)	104 (75.91 %)	173 (78.64 %)
SCC	158	115 (25.96 %)	29 (21.17 %)	43 (19.55 %)
Others	22	16 (3.61 %)	4 (2.92 %)	4 (1.81 %)

### 2.3 CT Examination and clinical features

CT images were acquired from the hospital using three different CT scanner models: Discovery CT 750 HD (GE Healthcare, United States), Somatom Sensation 64 (Siemens, Germany), and Brilliance iCT (Philips, Netherlands). Scanning parameters were as follows: 80–120 kVp, automatically modulated tube current, and a matrix of 512 × 512. Reconstruction protocols varied by manufacturer, GE CT systems: slice thickness of 1.25 mm with a 1.25 mm reconstruction interval. Philips and Siemens systems: slice thickness of 1 mm with a 1 mm reconstruction interval.

For the NLST cohort, CT images were acquired using eight scanner models: Aquilion (Canon Medical Systems, Japan); HiSpeed QX/i, LightSpeed Plus, LightSpeed QX/i, and LightSpeed 16 (GE Healthcare, United States); Mx8000 (Philips, Netherlands); and Sensation 16 and Volume Zoom (Siemens, Germany). Scanning parameters varied across sites, with tube voltage ranging from 80 to 140 kVp, tube current from 40 to 320 mA, and slice thickness between 2 and 5 mm.

For the NSCLC-Radiogenomics cohort, CT images were obtained using multiple scanners and acquisition protocols, with slice thickness ranging from 0.625 to 3 mm, tube current from 124 to 699 mA (mean: 220 mA), and tube voltage from 80 to 140 kVp (mean: 120 kVp).

Clinical information, including age, gender, and smoking history, was retrieved from the patient electronic medical record system. Two senior thoracic radiologists (with 9 and 15 years of experience in thoracic imaging, respectively) independently assessed the radiographic characteristics of the pulmonary lesions while blinded to the pathological results. The evaluated characteristics included: (1) lesion location; (2) mean diameter, calculated as the average of the longest and shortest axes of the lesion; (3) lesion density (pure ground-glass opacity, mixed density, or solid density); (4) clarity of the tumor-lung interface (clear or blurred); (5) lobulation; (6) spiculation; (7) pleural indentation; (8) vascular convergence; (9) vacuole sign; and (10) air bronchogram sign. Any disagreements during the evaluation were resolved through consultation between the two initial readers. Any persistent discrepancies were adjudicated by a third senior radiologist (with 23 years of experience in pulmonary imaging).

### 2.4 Tumor delineation and peritumor expansion

The regions of interest (ROIs) encompassing the primary GTV of each lesion were manually delineated on the original CT images by the two radiologists mentioned above. Discrepancies were resolved through consensus; in cases of persistent disagreement, a final decision was made by the third senior radiologist.

Both the original CT images and the corresponding ROI segmentation masks were resampled to an isotropic voxel size of 1 × 1 × 1 mmł using nearest-neighbor interpolation to ensure consistent spatial resolution and to improve feature extraction robustness. Following resampling, the left and right lung lobes were automatically segmented for each case using the TotalSegmentator module in 3D Slicer (version 5.7.0) (14).

Subsequently, all GTV ROIs were expanded isotropically by 3 mm margin (15) to generate the peritumoral area. This expansion was automatically corrected using the segmentation of lung lobes to ensure that the tumor expansion did not include the chest wall or other non-lung tissues. The delineation, resampling, and expansion of the tumor were all carried out using the 3D Slicer tool (version 5.7.0).

### 2.5 Habitat clustering and feature extraction

For each patient, the original GTV ROI and the expanded peritumoral region together defined the whole volume of interest (whole_VOI, WVOI). The voxel-based first-order entropy feature map for the WVOI was calculated using the PyRadiomics package (version 3.1) (16). The first-order entropy feature, defined by the Image Biomarker Standardization Initiative (IBSI) as Intensity Histogram Entropy (17), specifies the uncertainty or randomness in the image values. The formula for first-order entropy is presented in Eq. 1.


$$f\,i\,r\,s\,t\,o\,r\,d\,e\,r\,e\,n\,t\,r\,o\,p\,y=-\sum_{i=1}^{N_{g}}p\,\left(i\right)\,l\,o\,g_{2}\,\left(p\,\left(i\right)+\varepsilon \right)$$ (1)

Where (*p*_*i*_) is the normalized first-order histogram and equals **P**(_*i*_) /N_*p*_, in which **P**(_*i*_) is the first-order histogram with N_*g*_ discrete intensity levels, and N_*p*_ is the total number of voxels of WVOI. N_*g*_ is the number of non-zero bins (N_*g*_ = 25 in this study, commonly used for CT images). ε is an arbitrarily small positive number (≈ 2.2 × 10^–16^).

For each voxel in the WVOI, we created a super-voxel vector by combining the voxel’s gray level intensity with its first-order entropy. The K-means clustering algorithm was then applied to these super-voxel vectors to identify different subregions within the WVOI, thereby forming distinct habitats. To determine the optimal number of clusters (*k*) for *k*-means clustering, we analyzed all WVOIs using values of k set to 2, 3, and 4. For each value of *k*, the average Silhouette Score and the Davies-Bouldin Index (DB Index) were calculated. Both the Silhouette Score and DB Index are metrics used to evaluate clustering performance. The Silhouette Score ranges from -1 to 1, with higher values indicating better clustering results. Conversely, the DB Index measures the similarity between different clusters, with lower values (closer to zero) indicating better clustering performance.

**FIGURE 2:**
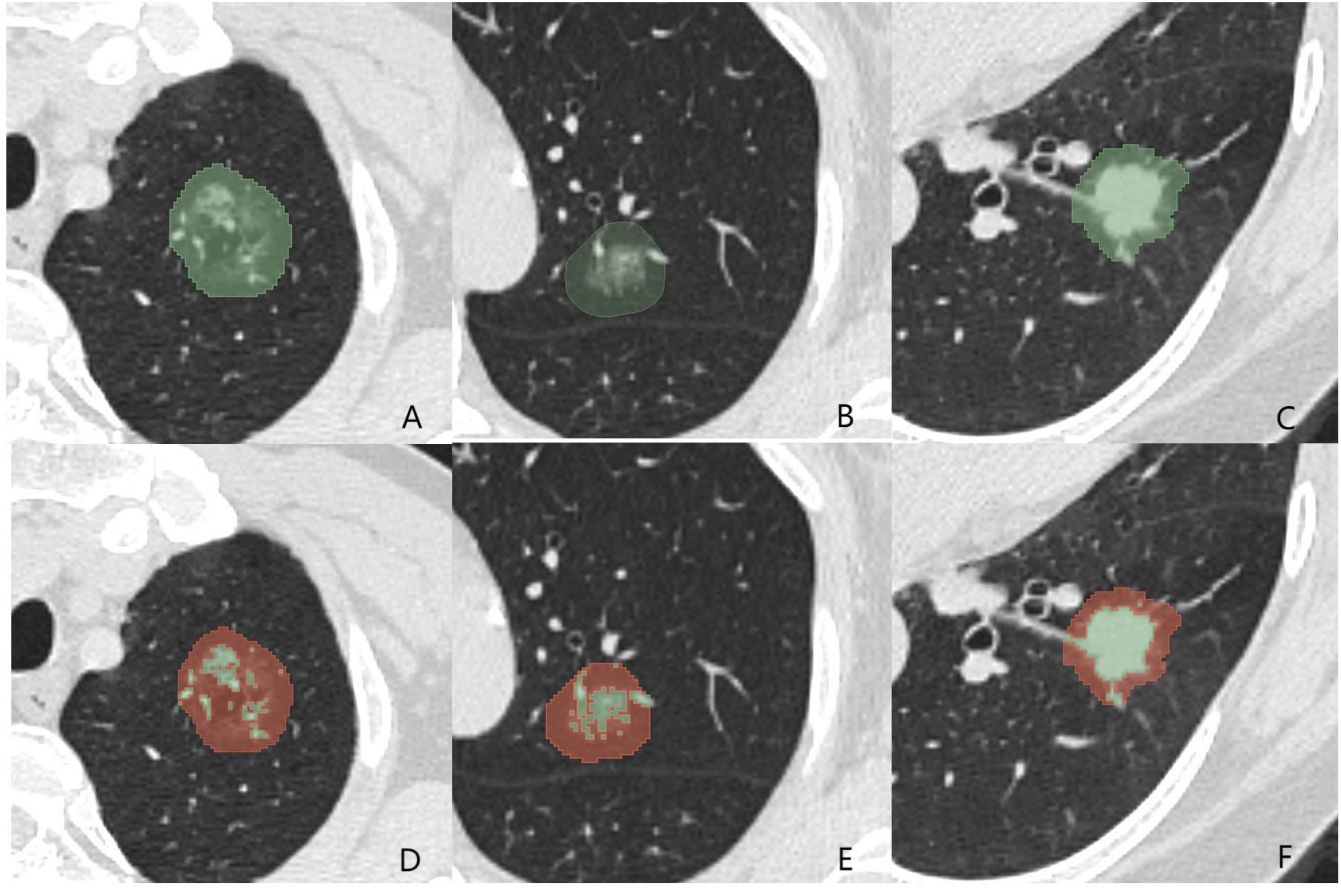
Examples of lung tumor delineation and habitats division with different grades. **(A)** Grade 1; **(B)** Grade 2; **(C)** Grade 3; **(D–F)** Habitat 1 (Green) and habitat 2 (Red) examples corresponding to **(A–C).**

Table 2 summarizes the average Silhouette Scores and DB Indices for different values of k. When *k* = 2, the highest Silhouette Score and the lowest Davies-Bouldin Index were observed simultaneously, indicating the best clustering performance among the three values of *k*. Therefore, value 2 of *k* was selected as the optimal number of clusters to apply to all patients. The two habitats identified through clustering were ranked based on voxel values, from high to low, and labeled as Habitat 1 and Habitat 2, respectively. Figure 2 illustrates the delineated WVOIs for three patients with pathological grades of Grade 1, Grade 2, and Grade 3, along with the two habitats derived from K-means clustering.

**TABLE 2:** Comparison of clustering metrics for various numbers of clusters.

The number of clusters	Silhouette Score (mean ± SD)	Davies-Bouldin Index (mean ± SD)
2	0.71 ± 0.02	0.43 ± 0.04
3	0.65 ± 0.02	0.48 ± 0.01
4	0.61 ± 0.01	0.50 ± 0.01

A total of 851 radiomics features were extracted from each of the WVOI, Habitat 1, and Habitat 2, respectively, using the PyRadiomics package (version 3.1). These features included 13 shape features, 19 first-order features, 75 texture features, and 744 wavelet-based high-order features. To address variability sources related to image acquisition and reconstruction among different cohorts, known as batch effects, the ComBat harmonization method was applied to harmonize radiomics features extracted from these three cohorts first. ComBat utilizes an empirical Bayesian framework to independently estimate and correct batch-specific mean and variance shifts for each feature while preserving true biological variation (18, 19). Harmonization was performed using the Python neuroComBat software (v0.2.9). Batch correction was applied separately to the concatenated radiomic datasets (WVOI, Habitat1, and Habitat2) for Model-1 and Model-2 with the following specifications: number of batches = 3, reference batch = NLST (largest sample size), no biological covariates adjusted, Empirical Bayes shrinkage enabled, and both location (mean) and scale (variance) adjustments applied.

### 2.6 Radiomics feature selection and model development

**FIGURE 3:**
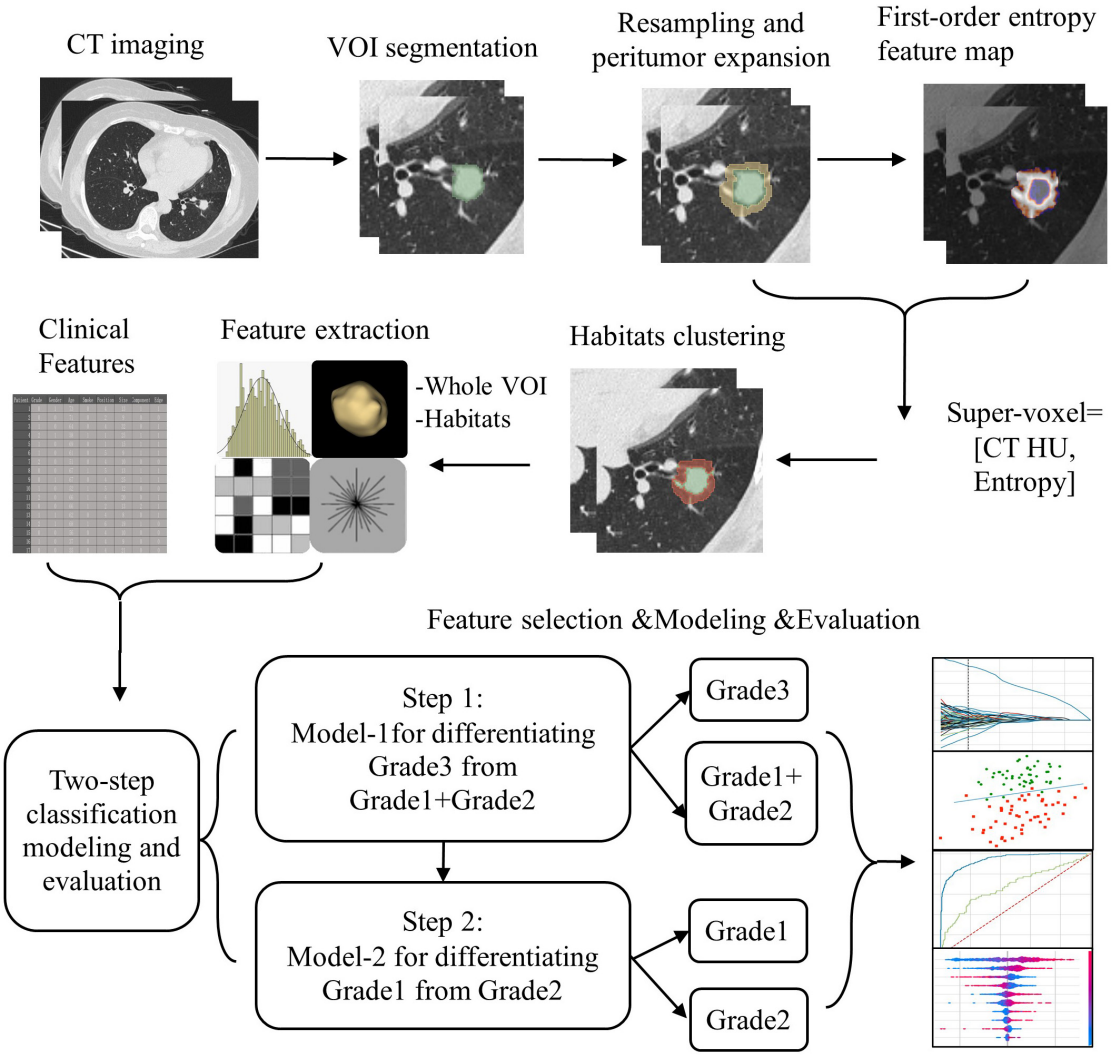
The workflow for model construction.

This study developed two-step binary classification models aimed at predicting the pathological grades of NSCLC. The first model, referred to as Model-1, was designed to distinguish between pathological Grade 3 and the combined Grades 1 and 2. The second model, referred to as Model-2, aimed to further differentiate between pathological Grade 1 and Grade 2. The workflow for model construction is illustrated in Figure 3.

**FIGURE 4:**
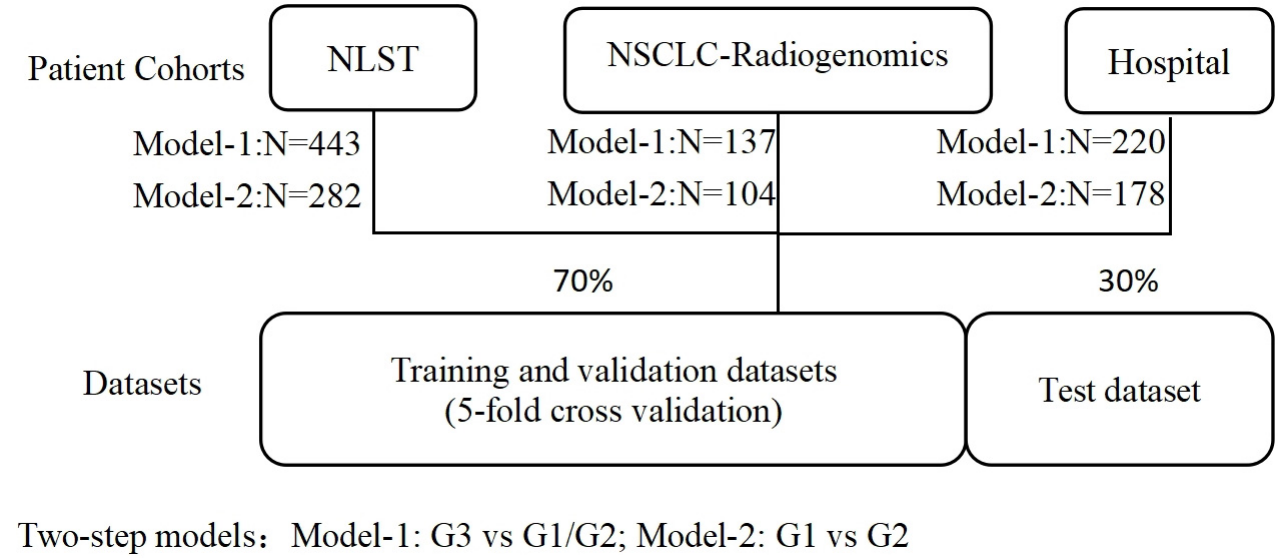
Train and test datasets division.

This study utilized samples from three cohorts to train both Model-1 and Model-2. The sample data were randomly stratified into training and testing sets in a 7:3 ratio during model training. Five-fold cross-validation was conducted to evaluate model performance and optimize hyperparameters, as shown in Figure 4.

Based on various combinations of radiomics features extracted from WVOI, Habitat 1, Habitat 2, and 13 clinical features, four classifiers were trained for both Model-1 and Model-2, respectively. The classifiers were as follows: (1) Clf WVOI, which utilized all radiomics features from WVOI only; (2) Clf Habitats, which included all radiomics features from Habitat 1 and Habitat 2 only; (3) Clf Clinical, which relied solely on the complete set of clinical features; and (4) Clf Total, which integrated all optimal features selected from the previous three single-modality models during the training process, as detailed in Table 3.

**TABLE 3:** Features composition and classifier definitions.

Name of classifier	Four classifiers to train with different feature			
	Clf WVOI	Clf Habitats	Clf Clinical	Clf total
Feature composition	Radiomics features of WVOI	Radiomics features of Habitat1+Habitat2	Clinical features	Total optimal features selected from the previous three models

Prior to training, outlier handling and Z-score standardization were applied to the radiomics feature data. These commonly used methods help reduce noise, equalize scales, and stabilize optimization, making the classification models more robust, reproducible, and trainable. To address the data imbalance issue in the training set, the synthetic minority over-sampling technique (SMOTE) was employed. Feature selection was performed primarily using the Least Absolute Shrinkage and Selection Operator (LASSO) and the Max-Relevance and Min-Redundancy (mRMR) algorithm. Finally, Logistic Regression (LR), a widely accepted traditional classification algorithm, was utilized to train the models. The mRMR was implemented using the mrmr_selection package (version 0.2.8). And outlier handling, Z-score, LASSO, and LR were all executed using scikit-learn (version 1.4.2).

### 2.7 Statistical analysis and classification performance evaluation

The independent samples *t*-test was utilized to compare normally distributed continuous variables between two groups; analysis of variance (ANOVA) was applied for comparisons among three or more groups. The Mann-Whitney U test was applied to continuous variables with non-normal distributions. The chi-square test was used for categorical variables, and the Kruskal-Wallis H test was used for ordinal variables. Spearman’s correlation was assessed for ordinal variables between two groups . A two-tailed *p* < 0.05 was considered statistically significant. All statistical analyses were conducted using Python 3.9 and R 4.2.

The performance of each classifier in the two-step classification models was evaluated using metrics such as the area under the receiver operating characteristic (ROC) curve (AUC), sensitivity (SENS), specificity (SPEC), and balanced accuracy (BACC) for an imbalanced dataset. BACC is calculated as the average of SENS and SPEC, ensuring that both categories contribute equally to the final prediction score in the binary classification. BACC provides a more reliable measure of model performance, particularly in the presence of class imbalance. Additionally, feature importances were analyzed using the SHAP (Shapley Additive Explanation) summary plot.

## 3 Results

### 3.1 Demographics and tumor characteristics

The demographic characteristics of the training and testing sets for Model-1 and Model-2 are summarized in Tables 4, 5, respectively. No significant differences were observed between the training set and testing set in terms of age, gender, smoking history, or tumor size.

**TABLE 4:** Demographics and tumor characteristics for Model-1 (G3 vs. G1/G2).

Variable	Subjects (N)	Train Set (*N* = 560)	Test Set (*N* = 240)	Statistics	*P*-value
Age (years, mean ± SD)	800	63.24 ± 7.69	64.31 ± 8.20	-1.763	0.078
**Gender, n (%)**					
Female	354	242 (43.21%)	112 (46.67%)	0.812	0.368
Male	446	318 (56.79%)	128 (53.33%)		
**Smoke, n (%)**					
No	385	273 (48.75%)	112 (46.67%)	0.292	0.589
Yes	415	287 (51.25%)	128 (53.33%)		
Tumor size (mm, mean ± SD)	800	21.63 ± 13.69	21.74 ± 12.62	-0.106	0.916

**TABLE 5:** Demographics and tumor characteristics for Model-2 (G1 vs. G2).

Variable	Subjects (N)	Train set (*N* = 394)	Test set (*N* = 170)	Statistics	*P*-Value
Age (years, mean ± SD)	564	63.17 ± 8.37	63.35 ± 7.75	-0.239	0.811
**Gender, n (%)**					
Female	278	193 (48.98%)	85 (50.00%)	0.049	0.825
Male	286	201 (51.02%)	85 (50.00%)		
**Smoke, n (%)**					
No	292	196 (49.75%)	90 (52.94%)	2.151	0.143
Yes	272	198 (50.25%)	80 (47.06%)		
Tumor size (mm, mean ± SD)	564	19.65 ± 10.40	19.34 ± 10.61	0.324	0.746

### 3.2 Correlation analysis of clinical features

The associations between the 13 clinical features and pathological grade were assessed using Spearman’s rank correlation method. As shown in Table 6, age, lesion position, and pleural indentation showed no significant correlation with pathological grade. In contrast, the other ten clinical features exhibited significant correlations. Notably, lesion density showed the strongest positive correlation (coefficient = 0.42, *p* < 0.001).

**TABLE 6:** Correlation analysis between Grade and clinical features.

Clinical features	Correlation coefficient	*P*-value
Age (Year)	0.05	0.17
Size (mm)	0.18	< 0.001
Gender (Female, Male)	0.14	< 0.001
Smoke (Yes, No)	0.12	< 0.001
Tumor-lung interface (Clear, blurred)	–0.25	< 0.001
Vacuole sign (Yes, No)	–0.13	< 0.001
Air bronchogram (Yes, No)	–0.18	< 0.001
Lobulation (Yes, No)	0.13	< 0.001
Spiculation (Yes, No)	0.23	< 0.001
Pleural indentation (Yes, No)	0.03	0.39
Vascular convergence (Yes, No)	0.08	0.02
Density (Ground glass, mixed, Solid)	0.42	< 0.001
Position (Left hilum, Left upper lobe, Left lower lobe, Right hilum, Right upper lobe, Right middle lobe, Right lower lobe)	–0.05	0.18

### 3.3 Radiomics modeling performance

#### 3.3.1 Performance of models

In Model-1 (G3 vs. G1/G2), the single-mode classifier Clf Habitats achieved the highest predictive performance among individual models, obtaining an AUC of 0.89 (95% CI: 0.86–0.93) and a BACC of 0.78 in the test set. This was followed by Clf_WVOI with an AUC of 0.83 (95% CI: 0.80–0.88) and a BACC of 0.75, whereas Clf Clinical performed the lowest performance, with an AUC of 0.79 (95% CI: 0.74–0.84) and a BACC of 0.75. Compared with single-modality classifiers, the multimodal integrated model (Clf Total), constructed from the optimal subset of WVOI, habitat, and clinical features, yielded superior predictive performance in the test set, with an AUC of 0.91 (95% CI: 0.87–0.94) and a BACC of 0.82.

**FIGURE 5:**
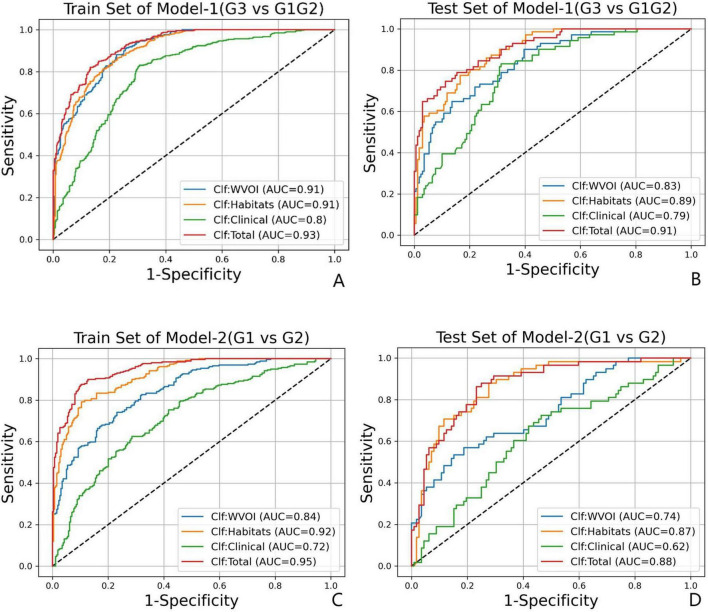
The ROC curves for Model-1(G3 vs. G1/G2) in **(A)** train set and **(B)** test set, and for Model-2(G1 vs. G2) in **(C)** train set and **(D)** test set.

In Model-2 (G1 vs. G2), Clf Habitats also significantly outperformed Clf WVOI and Clf Clinical, achieving an AUC of 0.87 (95% CI: 0.82–0.91) and a BACC of 0.79 in the test set. Clf Clinical again exhibited the lowest performance, with an AUC of 0.62 (95% CI: 0.55–0.70) and a BACC of 0.60. Clf Total achieved the highest performance in the test set, with an AUC of 0.88 (95% CI: 0.82–0.92) and a BACC of 0.81. The ROC curves and detailed performance metrics for all four classifiers in the training and test sets for both Model-1 and Model-2 are presented in Figure 5 and Table 7, respectively.

**TABLE 7:** Classification performance of two-step models.

Models	Classifier	Train set				Test set			
		AUC (95%CI)	Sensitivity	Specificity	Balanced accuracy	AUC (95%CI)	Sensitivity	Specificity	Balanced accuracy
Model-1	Clf WVOI	0.91 (0.89–0.93)	0.88	0.78	0.83	0.83 (0.80–0.88)	0.80	0.69	0.75
	Clf Habitats	0.91 (0.89–0.92)	0.85	0.79	0.82	0.89 (0.86–0.93)	0.83	0.73	0.78
	Clf Clinical	0.80 (0.78–0.83)	0.83	0.70	0.76	0.79 (0.74–0.84)	0.83	0.67	0.75
	Clf Total	0.93 (0.91–0.94)	0.82	0.87	0.84	0.91 (0.87–0.94)	0.80	0.84	0.82
Model-2	Clf WVOI	0.84 (0.81–0.87)	0.74	0.77	0.75	0.74 (0.67–0.81)	0.64	0.61	0.62
	Clf Habitats	0.92 (0.90–0.94)	0.79	0.90	0.84	0.87 (0.82–0.91)	0.84	0.73	0.79
	Clf Clinical	0.72 (0.68–0.76)	0.62	0.71	0.67	0.62 (0.55–0.70)	0.60	0.60	0.60
	Clf Total	0.95 (0.93–0.96)	0.87	0.90	0.89	0.88 (0.82–0.92)	0.86	0.77	0.81

**FIGURE 6:**
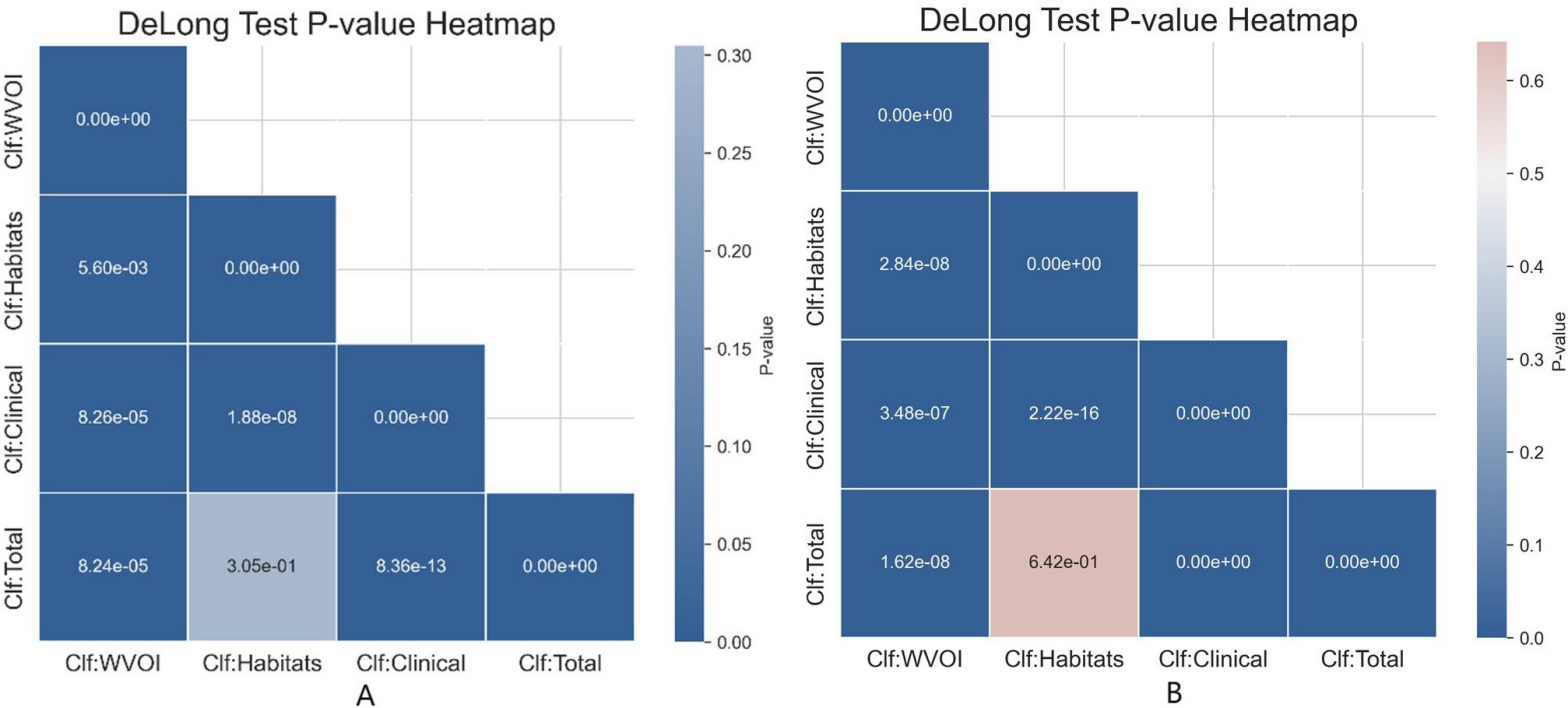
DeLong test *p*-value heatmap for **(A)** Model-1(G3 vs. G1/G2) and **(B)** Model-2(G1 vs. G2).

The DeLong test was used to compare the classification performance of the four classifiers in the test sets of Model-1 and Model-2. The results demonstrated that, in both Model-1 and Model-2, Clf Habitats significantly outperformed Clf WVOI and Clf Clinical (*P* < 0.001), whereas no statistically significant difference was observed between Clf Habitats and Clf Total. The comparative results are illustrated in Figure 6.

#### 3.3.2 Interpreting Clf total model decisions via SHAP

**FIGURE 7:**
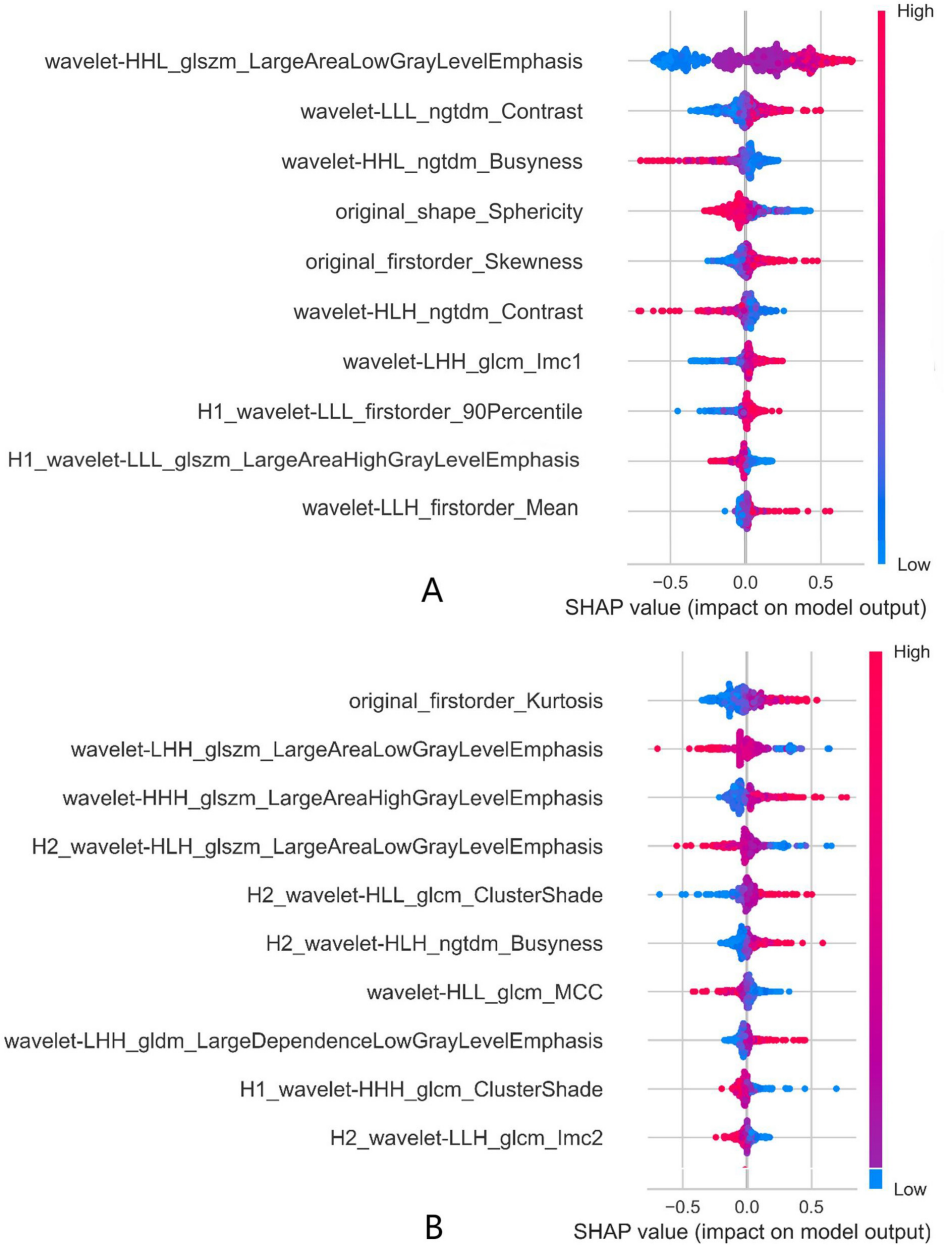
SHAP feature importance analysis for Clf Total of **(A)** Model-1(G3 vs. G1/G2) and **(B)** Model-2(G1 vs. G2) (top 10 features shown); prefix of H1 and H2 stand for Habitat1 and Habitat2.

SHAP summary plots were utilized to interpret the decision-making process of the overall classifier (Clf Total) in Model-1 and Model-2. Figure 7 displays the top ten most contributory features and quantifies the marginal contribution of each feature to the model output. In both models, texture features and high-order features derived from wavelet transformations constituted the majority of the feature weights. In Model-1, wavelet-HHL_glszm_LargeAreaLowGrayLevelEmphasis provided the greatest predictive contribution to Clf Total, whereas original_firstorder_Kurtosis was the most contributory feature in Model-2. Habitat-related features occupied two of the top feature positions in Model-1; this number increased to five in Model-2.

## 4 Discussion

The imaging features of NSCLC correlate with tumor grade: G3 tumors show distinct characteristics relative to G1, whereas G2 presents intermediate features that often lead to misclassification as either G1 or G3. To overcome this diagnostic challenge, we designed a two-step binary classification framework. Model-1 classified G3 (positive) against combined G1/G2 (negative). Model-2 then differentiated G1 from G2. The results confirm the viability of this strategy. The stepwise design aligns with clinical decision-making, thereby improving model interpretability and acceptability.

Previous studies have demonstrated the value of radiomics in predicting the pathological grade of LUAD. For instance, Wang et al. (11) developed a radiomics-deep learning model to identify micropapillary/solid components, achieving an overall accuracy of 0.913. Ninomiya K. et al. (20) used high-resolution CT-based radiomics to predict solid and micropapillary components with an AUC of 0.902. However, these models only identify high-grade (G3) tumors and do not differentiate between G1 and G2 grades. Moreover, their study populations were restricted to LUAD, excluding other NSCLC subtypes. Another limitation is that their feature extraction methods may not adequately capture intratumoral spatial heterogeneity, which constrains the generalizability and clinical utility of these models. In contrast, habitat analysis employs unsupervised clustering to partition tumors into distinct habitats based on their imaging phenotypes. These habitats correspond to divergent proliferative, invasive, and metabolic profiles, which may predict variations in treatment response. Consequently, this approach yields more granular biomarkers, improving its potential to support precision diagnostics and guide therapy planning (7, 21, 22).

This study investigates habitat radiomics to predict pathological grading in NSCLC. The Clf Habitats showed advantages in single-modality tasks, achieving AUC values of 0.89 for Model-1 and 0.87 for Model-2, outperforming the WVOI and clinical models. These results highlight the superior predictive value of the habitat radiomics model for NSCLC pathological grading. The multimodal model (Clf Total) integrating WVOI, habitat, and clinical features achieved AUC values of 0.91 for G3 prediction and 0.88 for G1. While Clf Habitats exhibited high sensitivity, its specificity (0.73 for both Model-1 and Model-2) was lower than Clf Total (0.84 for Model-1, 0.77 for Model-2), making Clf Total more suitable for preoperative scenarios where controlling false positives is critical. Conversely, the simpler Clf Habitats model, with reduced data complexity and high sensitivity, may be more applicable for screening purposes where identifying all potential positive cases is a priority.

SHAP analysis revealed that the most predictive features were predominantly derived from wavelet-transformed texture metrics. In Model-1, the feature wavelet-HHL_glszm_LargeAreaLowGrayLevelEmphasis was the most influential predictor for classifying G3 tumors. This metric quantifies the predominance of large, interconnected regions with low signal intensity. Higher values indicate more extensive hypointense areas on imaging, which are highly suggestive of pathological findings such as necrosis—a well-documented characteristic of poorly differentiated tumors compared to their well-differentiated counterparts (23, 24). In Model-1, two habitat-based features ranked among the top 10 most important features. In contrast, in the more challenging task of Model-2, the contribution of habitat features increased significantly, with five such features ranking the top 10. Moreover, their SHAP absolute values were higher than those of habitat features in Model-1. This discrepancy suggests that spatial tumor heterogeneity may play a more critical role in distinguishing between G1 and G2 grades, while habitat features provide greater discriminatory power in clinical scenarios requiring subtle differentiation between pathological grades.

Our two-stage framework is supported by prior evidence highlighting the intrinsic complexity and heterogeneity of pathological grading in lung cancer. Histopathological studies have shown that multiple growth patterns and differentiation grades frequently coexist within the same tumor, particularly in intermediate-grade categories, resulting in substantial interobserver variability and limited reproducibility when grading is performed using a single-step strategy (4, 24). In line with this observation, Zheng et al. (25) proposed a two-step radiomics framework for IASLC grading of invasive pulmonary adenocarcinoma, in which an initial submodel identified the presence of any high-grade component, followed by a second submodel for predominant subtype differentiation. This staged design improved discrimination of higher-grade lesions compared with a one-step model. Furthermore, the superior performance of the habitat-based model indicates that such a two-step strategy effectively refines the accuracy of pathological grading. Most earlier radiomics studies addressing lung cancer grading have focused on adenocarcinoma and have relied on binary classification strategies to detect specific high-risk growth patterns, such as micropapillary or solid components (26–28). Although these approaches demonstrated reasonable accuracy when features were extracted from near-pure histopathological regions, they implicitly assume spatial homogeneity or require prior knowledge of subtype-dominant areas. Consequently, their applicability to tumors with mixed or ambiguous histology, or to broader NSCLC populations encompassing multiple histologic subtypes, remains limited.

Habitat-based radiomics provides a structured solution to this limitation by explicitly delineating spatially distinct intratumoral subregions based on voxel-wise radiomic patterns. Bernatowicz et al. (29) demonstrated that voxel-wise radiomics features characterizing texture heterogeneity, particularly entropy- and energy-based metrics, can be reproducibly computed across lung cancer CT datasets and yield stable imaging habitats when robust features are selected. These findings establish a methodological foundation for capturing biologically meaningful spatial heterogeneity beyond whole-tumor summary statistics. Recent NSCLC studies further support the clinical relevance of heterogeneity-aware imaging biomarkers, with habitat-based approaches improving prediction of recurrence, treatment response, and immune-related outcomes when integrated with complementary molecular or clinical data (10, 21, 30). Collectively, these results indicate that spatially resolved imaging phenotypes capture biologically relevant information lost in global analyses.

Recent advancements in molecular biology, immunology, and nanotherapy have underscored that NSCLC exhibits pronounced multidimensional heterogeneity across spatial, cellular, and molecular scales (31–37). Variations in receptor expression patterns, the extent of immune infiltration, and drug sensitivity collectively manifest the biological diversity within individual tumors and across patient cohorts, fundamentally influencing therapeutic outcomes and clinical prognosis. These insights align closely with the understanding of functional regional heterogeneity observed at the imaging level, suggesting that distinct intra-tumoral functional domains can be visualized and quantified via radiomics. Consequently, habitat-based radiomics establishes a robust conceptual bridge between biological complexity and macro-scale imaging phenotypes, facilitating the non-invasive evaluation of spatially heterogeneous tumor biology.

This study has several limitations. First, this study incorporated CT images from multiple centers and public datasets, resulting in heterogeneity in scanner models, acquisition protocols, and reconstruction parameters. Although ComBat harmonization was applied to mitigate batch effects, residual variability related to underlying physical imaging characteristics and reconstruction algorithms cannot be fully eliminated, which may influence radiomics feature stability and model generalizability. Second, the model was developed and validated using retrospective data, which may introduce selection bias. The two public datasets used also reflect imaging acquired over an earlier time period. Future studies may incorporate prospective, multi-center data for external validation to further improve model generalizability. Third, pathological grading of NSCLC was predicted based solely on non-contrast CT images. Future work may integrate additional imaging modalities, such as contrast-enhanced CT or spectral imaging, to improve predictive performance. Fourth, the clinical semantic model included only CT features and basic clinical parameters. In addition, this study did not include benchmarking against multiple alternative classifiers. The focus of this work was on assessing a two-stage, heterogeneity-aware framework rather than on algorithmic comparison, and direct classifier benchmarking is inherently confounded by differences in data composition, feature engineering, and grading definitions. Therefore, such comparisons were considered beyond the scope of the present study.

## 5 Conclusion

Habitat radiomics offers significant advantages over traditional radiomics in predicting the pathological grading of NSCLC by quantifying tumor spatial heterogeneity. The multimodal model developed in this study shows strong classification performance and greater specificity, providing essential evidence for developing personalized treatment strategies for NSCLC patients.

## Data Availability

The original contributions presented in the study are included in the article/supplementary material, further inquiries can be directed to the corresponding author.

## Footnotes

1
https://www.cancerimagingarchive.net/collection/nlst/

2
https://www.cancerimagingarchive.net/collection/nsclc- radiogenomics/
